# Bacteriophage–Antibiotic Combination Therapy against *Pseudomonas aeruginosa*

**DOI:** 10.3390/antibiotics12071089

**Published:** 2023-06-22

**Authors:** Guillermo Santamaría-Corral, Abrar Senhaji-Kacha, Antonio Broncano-Lavado, Jaime Esteban, Meritxell García-Quintanilla

**Affiliations:** 1 Department of Clinical Microbiology, IIS-Fundación Jiménez Díaz, Av. Reyes Católicos 2, 28040 Madrid, Spain; 2 CIBERINFEC—Infectious Diseases CIBER, 28029 Madrid, Spain

**Keywords:** *Pseudomonas aeruginosa*, treatment, bacteriophage, combination

## Abstract

Phage therapy is an alternative therapy that is being used as the last resource against infections caused by multidrug-resistant bacteria after the failure of standard treatments. *Pseudomonas aeruginosa* can cause pneumonia, septicemia, urinary tract, and surgery site infections mainly in immunocompromised people, although it can cause infections in many different patient profiles. Cystic fibrosis patients are particularly vulnerable. In vitro and in vivo studies of phage therapy against *P. aeruginosa* include both bacteriophages alone and combined with antibiotics. However, the former is the most promising strategy utilized in clinical infections. This review summarizes the recent studies of phage-antibiotic combinations, highlighting the synergistic effects of in vitro and in vivo experiments and successful treatments in patients.

## 1. Introduction

*Pseudomonas aeruginosa* is a Gram-negative bacillus, widely distributed in the environment, that causes important infections, generally as an opportunistic pathogen [1,2,3]. This bacterium is associated with high morbidity and mortality in patients with underlying pathology, such as cystic fibrosis (CF), chronic obstructive pulmonary disease (COPD), and bronchiectasis, among other pulmonary diseases [2,3]. This opportunistic pathogen is one of the causal agents of ventilator-associated pneumonia (VAP), since it can colonize hospital equipment, saline solution, and soap. The treatment of this pathogen is very diverse depending on the pathology in which it is involved. Among the drugs that are most used against this bacterium are ceftazidime, amikacin, colistin, and meropenem. Monotherapy and dual therapy regimens are established based on the antibiotic susceptibility that is present and the type of pathology. The resistance mechanisms presented by this bacterium make it challenging to establishing an effective treatment for *P. aeruginosa* [4]. In recent decades there has been a notable increase in infections by this bacterium, especially by multidrug-resistant (MDR) and extensively drug-resistant (XDR) clones [5,6]. The continued use of antibiotics, the increase in the prevalence of chronic diseases (especially respiratory), and the use of immunosuppressants have resulted in an increase in infections by this pathogen and adverse consequences in terms of the morbidity and mortality of these patients [7].

MDR and XDR *P. aeruginosa* bacteria exhibit varied resistance mechanisms acquired by chromosomal mutations or by horizontal transmission of genetic material. Among these resistance mechanisms, we can find the production of β-lactamases of the AmpC type, in addition to the fact that the outer membrane of this bacterium has extremely low permeability; this confers innate resistance to many antimicrobials and, in turn, the membrane itself is capable of expressing porins and active expulsion systems such as MexAB-OprM, MexCD-OprJ, MexEF-OprN and MexXY-OprM [8,9]. These systems can affect antimicrobials such as beta-lactams, fluoroquinolones, and aminoglycosides. Regarding the mechanisms of horizontal transmission, this bacterium can encode different modifying enzymes that can act against many antimicrobials; among them, we can mention the production of β-lactamases of extended-spectrum (BLEE), carbapenemases, and aminoglycoside-modifying enzymes [10]. Other species of the genus *Pseudomonas* and also of the genus *Acinetobacter* act as environmental reservoirs of antimicrobial resistance systems of *P. aeruginosa* that may be encoded in integron cassettes and be transmitted to *P. aeruginosa* [3]. Regarding its virulence factors, *P. aeruginosa* presents a wide variety, which contribute to its pathogenicity. In its external membrane, there is the lipopolysaccharide LPS common with other Gram-negative bacilli with endotoxic activity [11] and several porins, such as the OprF, OprH, and OprD Superfamilies [12]. On the other hand, it is important to highlight their high capacity to form biofilms, which increase the resistance not only to antimicrobial but also to inhospitable environmental factors [13]. Among the mucoid substances they produce in their biofilms, the most studied is alginate [14]. Finally, other factors such as type IV pili, flagellum, and numerous secretion systems as well as secondary metabolites such as pyocyanin can be highlighted, the latter being responsible for aggravating pulmonary diseases due to its pro-inflammatory activity and oxidative damage mediated by the formation of oxygen free radicals [15]. All these virulence factors allow *P. aeruginosa* to persist in vulnerable patients with various chronic pathologies, worsening their prognosis and causing persistent infections or recurrences whose treatment is a clinical challenge [7].

All these factors contribute to the need to search for new therapeutic alternatives, among which phage therapy can be highlighted. Bacteriophages capable of infecting *P. aeruginosa* have been isolated since the mid-20th century, and this therapeutic tool, combined with antimicrobials, may be extremely useful in the treatment of unresolved *P. aeruginosa* infections.

Several studies have demonstrated the efficacy of bacteriophages against *P. aeruginosa*, especially in combination with antibiotics. In vitro studies show that the use of temperate phages (such as HK97) in combination with suboptimal concentrations of ciprofloxacin can drastically reduce the population of *P. aeruginosa*; these studies have been carried out using assays in agar plates as well as in microtiter plates, in which the bacteria were exposed against serial concentrations of antibiotics as well as different dilutions of phage [16]. In turn, lytic bacteriophages can serve as adjuvants in conjunction with antibiotics by reducing the MICs of antibiotics in such a way as to increase susceptibility to antibiotics that might previously have been unsuitable, although this phenomenon is highly dependent on the mechanisms of action of the antibiotics administered in conjunction with the phages. These assays are carried out in microtiter plates in which inocula with a determined concentration of bacteria were added and exposed to both the presence of bacteriophages and antibiotics, after which successive absorbance measurements were taken at 600 nm every 15 min for 24 h at 37 °C under shaking conditions [17].

The use of phages combined with antibiotics is the most realistic way of applying this therapy to patients to avoid the appearance of resistance and achieve greater therapeutic success. Phages have recently regained interest in the fight against antibiotic multi-resistance. They are safe, although their effectiveness is highly dependent on the strain against which they are applied [18]. There remains a lack of knowledge regarding the use of bacteriophages, including information on their influence on the immune response of patients as well as their production and processing for administration in; thus, their use is currently limited to clinical trials and compassionate use situations [18]. In order to be useful for clinical practice, in this paper we review the most relevant work of the last five years based on the combination of bacteriophages and antimicrobials against *P. aeruginosa* infections. We describe in vitro and in vivo studies as well as case reports.

## 2. In Vitro Models

Bacteriophage–antibiotic combination in vitro therapy against multidrug-resistant (MDR) *P. aeruginosa* has been demonstrated with almost all antibiotics commercially available: aminoglycosides (amikacin, gentamycin, streptomycin, and tobramycin), β-lactams (ceftazidime, ceftriaxone, meropenem, and piperacillin), fluoroquinolones (ciprofloxacin), fosfomycin, macrolides (erythromycin), polymyxins (colistin and polymyxin B) and tetracyclines. Furthermore, several researchers have tried to optimize a checkerboard method to determine bacteriophage–antibiotic interactions and to determine whether synergy can be obtained with both simultaneous and successive application of these antibacterial agents [19].

In vitro treatment with bacteriophages and antibiotics has been able to significantly increase susceptibility and re-sensitization of MDR *P. aeruginosa* strains to antibiotics [20,21,22,23,24,25,26,27,28,29,30,31]. All these studies were performed by conducting assays to determine the minimum inhibitory concentration (MIC) for antibiotics and bacteriophages according to the Clinical and Laboratory Standards Institute (CLSI) broth microdilution protocol and the fractional inhibitory concentrations (FIC) of antibiotics in the presence of phages using the checkerboard method [32]. However, this bacteriophage–antibiotic synergy can be inhibited by the competition between temperate and chronic viruses, as Landa et al. demonstrated [33]. Chronic viruses can trigger the production and release of new viruses by the host cell without killing it but also have a latent cycle in which its genetic material is embedded into the bacterium’s genome [34,35]. Meanwhile, temperate viruses have a lytic cycle, in which virus production by bacteria bursts out of the host cell, and a latent cycle, where the virus remains inactive in the bacteria until induced to replicate. The authors modeled the synergy between antibiotics and these two viral types in controlling bacterial infections. While combinations of antibiotic and temperate viruses exhibited synergy, the combination of temperate and chronic viruses inhibited antibiotic control of bacteria. Antibiotics had the highest effect on the bacterial population infected with temperate viruses, since latent bacteria were induced by antibiotics into the lytic cycle. When chronic and temperate viruses were present in the absence of antibiotics, the temperate viruses could still lyse the bacteria. Otherwise, when the concentration of antibiotics was low, the presence of both viruses had a larger negative impact on the bacterial population than when only chronic viruses were present. Nevertheless, at higher concentrations of antibiotics the bacterial populations become equivalent to the effect when only chronic phages are present. The two populations converge because chronic viruses out-compete temperate viruses due to the stress-induced chronic virus production rate increase.

Ciprofloxacin has been the most utilized antibiotic combined with bacteriophages [36,37,38,39]. The impact of this antibiotic (as colistin) on the bactericidal, bacteriolytic, and new virion production of *P. aeruginosa* bacteriophages was assessed in a recent study by optical density-based “lysis profile” assays in the presence and absence of antibiotics [40]. Lysis profiles require the addition of bacteriophages at high bacterial densities, as the impact on the bacteria population is observed as a reduction of the turbidity of the bacterial culture. Colistin was shown to substantially interfere with bacteriolytic and virion-production activities; the bacteriophage utilizing LPS as its surface receptor could be a contributor to the observed antagonism of this bacteriophage infection activity by colistin, as LPS is directly disrupted by that antibiotic. In adsorption experiments, phage virion-attachment antagonism was observed in the presence of colistin (MIC). In contrast to the colistin results, negative impacts on lysis-profile kinetics are minimal with ciprofloxacin. Ciprofloxacin had no negative impact on phage adsorption rates even at high concentrations. These results suggest that ciprofloxacin could be useful as a concurrent phage therapy co-treatment, especially when phage replication is required for treatment success.

Bacteriophage PEV20 synergistic effects with ciprofloxacin has been proven in several studies [41,42,43] to enhance eradication of *P. aeruginosa* biofilm associated with cystic fibrosis and wound patients [43]. In addition, reducing the antibiotic concentration required to fight against these bacterial infections is associated with biofilms in these patients. These results were assessed by quantification of biofilm biomass, viability, and determination of minimum biofilm inhibitory concentration (MBIC). Furthermore, the antimicrobial effect of nebulized PEV20 with ciprofloxacin was determined against *P. aeruginosa* strains isolated from sputum CF patients by assessing bacterial killing and performing time-kill studies [42].

Conversely, a recent study demonstrated that combined bacteriophage and antibiotic pretreatment with ciprofloxacin and two phages (vB_PaeP_4024 and vB_PaeS_4069) prevents *P. aeruginosa* infection of wild type and CFTR epithelial cells and the emergence of bacteriophage-resistant mutants without inducing an inflammatory response, while administration of single bacteriophages, phage cocktails, or ciprofloxacin led to development of bacterial regrowth due to phage-resistant mutants [44]. In an innovative study, Ferran et al. simulated oral treatment with ciprofloxacin and phage-inhaled administration in *P. aeruginosa* respiratory infections [45]. Antibiotic in vitro treatment reproduced a maximum concentration of 1.5 µg/mL and a half-life of 4 h. Ciprofloxacin and bacteriophage single treatment generated resistant bacteria in less than 30 h. However, the combination of bacteriophages with ciprofloxacin was able to prevent the growth of resistant bacteria as simultaneous and delayed treatment. To assess the robustness of the combined treatment, the Hollow Fiber Infection Model (HFIM) was inoculated with a 1000-fold higher bacterial inoculum, while the regimen of either ciprofloxacin and phages at a Multiplicity of Infection (MOI) of 0.1 was the same. Simultaneous administration of combined treatment quickly decreased bacterial density below the limit of detection (LOD) but increased again after reducing susceptibility to ciprofloxacin (16- to 32-fold higher MIC) and bacteriophages compared to the naïve population. In contrast, in the delayed treatment, the initial reduction of bacteria was slower, with bacterial density falling below the LOD at 1 h for one replicate and 6 h for the other. However, after this decline, no increase in the bacterial density was observed, and gain no colony could be recovered on samples taken during the next 72 h. The authors concluded that when phages reduce the size of the bacterial population, the remaining population is not sufficient to include less-susceptible mutants to ciprofloxacin.

The synergistic action of bacteriophages and antibiotics has also been studied against dual-species biofilm, such as *P. aeruginosa–S. aureus* biofilm. Akturk et al. described the synergistic action of phages and antibiotics (ciprofloxacin, gentamicin, and meropenem) on 48 h *P. aeruginosa–S. aureus* biofilm when treated in simultaneous or sequential combination [46]. Phage or antibiotic single treatment developed a moderate effect on biofilm; however, when applied simultaneously, the effect was extensively improved. In addition, when gentamicin and ciprofloxacin were administered sequentially 6 h after phage treatment, a remarkable biofilm diminution was noticed, exhibiting even eradication of the biofilm. Furthermore, it was determined that the antibiofilm effect depends only on antibiotic concentration, not on its type: almost complete biofilm eradication was observed only when antibiotic concentration was higher or equal to MIC. Otherwise, achieving a similar gentamicin antibacterial effect on *P. aeruginosa*–*S. aureus* biofilm required increase of the antibiotic concentration: bacteriophage–gentamicin 8xMIC sequential administration nearly eradicated the *P. aeruginosa* population and was the most effective treatment on the *S. aureus* population.

Tkhilaishvili et al. demonstrated the potential use of combined bacteriophages Sb-1 and PYO with antibiotics for killing dual-species biofilm formed by *P. aeruginosa* and methicillin-resistant *S. aureus* (MRSA) [47]. They also investigated the effect of either simultaneous or staggered application of commercially available bacteriophages (Pyophage and Staphylococcal bacteriophage) and ciprofloxacin against dual-species biofilm in vitro. In this experiment, biofilms were formed in porous glass beads, and different techniques (microcalorimetry, sonication, and electron microscopy) were applied for assessing the anti-biofilm properties of treatments. Antibiotics tested alone against biofilms required high concentrations ranging from 256 to 512 µg/mL to show an inhibitory effect, whereas bacteriophage alone showed good and moderate activity against MRSA biofilms and dual-species biofilms, respectively, but low activity against *P. aeruginosa* biofilms. The combination of antibiotics and bacteriophages showed a remarkable improvement in the anti-biofilm activity of both antimicrobials with complete eradication of dual-species after staggered exposure to Pyophage or Pyophage+Staphylococcal phage for 12 h followed by 1 µg/mL of ciprofloxacin, a dose achievable by intravenous or oral antibiotic administration.

Lastly, Monahar et al. established the first approach to study the potential therapeutic approach of using bacteriophage–antibiotic combinations for treating infections caused by *P. aeruginosa* and *Candida albicans* [39]. Bacteriophage–fluconazole treatment was effective against 6-h-old dual-species biofilm, but not against 24-h-old biofilms. Likewise, the combination of antibiotics with the bacteriophage showed no synergistic effect on dual-biofilm.

## 3. In Vivo Models

The in vivo models described in the scientific literature testing the effect of combinations of bacteriophages and antibiotics are scarce (Table 1).

Regarding the lung infection in vivo models, Lin et al. demonstrated the in vivo effect of an inhalable powder of co-spray drying *Pseudomonas aeruginosa* phage PEV20 with ciprofloxacin using a neutropenic model of acute lung infection [48]. Firstly, the clinical *P. aeruginosa* (resistant to ciprofloxacin, aztreonam, and amikacin) was sprayed directly into the trachea using a micro sprayer. The powders (1 mg) of single ciprofloxacin (0.33 mg), single PEV20 (10^6^ PFU/mg), and the combination were aerosolized into the trachea of anesthetized mice using a dry powder insufflator. Intratracheally treatment with PEV20–ciprofloxacin combination powder significantly reduced the bacterial load in mice lungs by 5.9 log_10_, whereas single treatments with phage and antibiotics failed to reduce the burden. The efficacy was synergistic, as the observed killing effect for the combination powder was statistically higher than the additive effect of single treatments, with both showing nil effect at 24 h. Assessment of immunological responses in the lungs showed reduced inflammation associated with the bactericidal effect of PEV20–ciprofloxacin powder. This study represents the first proof-of-concept study demonstrating the synergistic efficacy of combined phage–antibiotic powder treatment in a mouse lung infection model.

In addition, Duplessis et al. described the IP administration of a three-bacteriophage cocktail with/without meropenem in an acute immunocompromised mouse model of MDR *P. aeruginosa* pulmonary infection [49]. Firstly, they assessed the potential therapeutic IP administration of the bacteriophage cocktail (10^9^ PFU/mL) alone for 120 h, delayed relative to bacterial inoculation by 3 h. IP administration of phage cocktails did not protect mice from death. Lastly, they assessed if subcutaneous administration of meropenem at subinhibitory concentrations could enhance bacteriophage efficacy, delayed by 3 h relative to bacterial inoculation. The combined treatment of meropenem and phage significantly enhanced therapeutic protection against pulmonary infection and significantly reduced bacterial burden in the lungs and spleen. These data support that phage-administered IP can penetrate the pulmonary tissues and, in combination with a sub-efficacious dose of antibiotic, can slow bacterial proliferation but not protect against a lethal outcome.

Cafora et al. tested the effects of combining bacteriophage therapy (four-phage cocktail) and antibiotic treatment (ciprofloxacin) against *P. aeruginosa* infections in an innovative cystic fibrosis zebrafish model [50]. Zebrafish CFTR channels present a similar structure to human CFTR. Additionally, zebrafish CFTR knockdown presents susceptibility to *P. aeruginosa* infections. As bacteriophage therapy, the authors injected 300–500 PFU/embryo of phage cocktail into the yolk sac of CF+PAO1-infected embryos. In the case of antibiotic treatment, it was done by incubation of CF+PAO1 embryos in fish water containing 100 µL of ciprofloxacin. Antibiotic treatment reduced lethality in comparison to CF+PAO1 embryos. Interestingly, combined treatment with phages and ciprofloxacin enhanced the reduction of lethality compared to every single treatment.

Finally, Engeman et al. described the synergistic killing of *P. aeruginosa* by phage–antibiotic combination treatment in a mouse dorsal wound model [20]. Mice were wounded dorsally, infected with PA01::lux, and treated with a PAM2H bacteriophage cocktail (10^8^ PFU/mL topically on the wound once a day and PBS intraperitoneally twice a day), ceftazidime (CAZ) (PBS topically on the wound once a day and CAZ intraperitoneally twice a day), or PAM2H and CAZ in combination (10^8^ PFU/mL topically on the wound once a day and CAZ intraperitoneally twice a day). Treatment with PAM2H in combination with CAZ resulted in a synergistic reduction in bacterial burden in vivo. Reduced virulence was noticed in the bacteria recovered from post-treated mice wounds in a larvae model.

## 4. Case Reports

In the majority of clinical cases of *P. aeruginosa* infections, bacteriophages have not been administered as a single treatment they have been applied concomitantly with antibiotics as an adjuvant treatment. Hereafter, we outline a series of clinical cases in which compassionate use with bacteriophage or a cocktail of phages were administered (intravenously, locally, or nebulized) concomitantly with antibiotics as an adjuvant treatment against *P. aeruginosa* with clinical resolution of different infections, mainly chronic (Table 2).

Ferry et al. described several cases in which adjuvant bacteriophage therapy was necessary to treat *P. aeruginosa* infections. One of them was an 88-year-old male patient with prosthetic joint infection (PJI) of the knee caused by ceftazidime and ciprofloxacin susceptible to *P. aeruginosa* [51]. As conventional treatment with antibiotics (IV ceftazidime and oral ciprofloxacin) was not effective and prosthesis explantation or exchange was not suitable, phage therapy was established as an adjuvant treatment to try to control the infection. As bacteriophage therapy was utilized, three phages in a cocktail (10^9^ PFU/mL) were administered through the arthroscope after conventional arthroscopy. After receiving bacteriophages and antibiotics, the patient rapidly showed signs of improvement.

They also described the case of a 74-year-old man with melanoma who experienced catheter-related bacteremia due to multidrug-resistant *P. aeruginosa* in December 2017, treated successfully with colistin and meropenem [52]. He was diagnosed with a spinal abscess in December 2018, and the aspiration revealed a pandrug-resistant *P. aeruginosa,* resistant to all antibiotics. Antibiotic treatment (colistin and rifampicin) rapidly ceased as a consequence of nephrotoxicity and ineffectiveness. The medical team proposed phage–antibiotic treatment combined with a surgical staged strategy. The first stage consisted of a spinal surgical procedure with local administration of a three-phage cocktail (10^6^ PFU/mL) and IV cefiderocol for 6 weeks. Two weeks after the end of the first stage and 2 weeks after the withdrawal of cefiderocol, the second stage was performed, with local administration of a phage cocktail before inserting the intersomatic cages at L2–L3 and L3–L4 levels. Cefiderocol was started again intravenously, pending the culture results. However, *P. aeruginosa* still grew in cultures from the bone biopsy, with a small colony variant phenotype susceptible to bacteriophage cocktail and cefiderocol. Although the strain had become resistant to this antibiotic, colistin was added intravenously to potentially synergize with cefiderocol. As the cultures still revealed the persistence of *P. aeruginosa*, a phage cocktail was also added intravenously over 3-hour infusions every day for 21 days. Antibiotics were stopped at 3 months. The outcome of the patient was favorable during the follow-up of 21 months, without implant loosening nor clinical signs of infection, and the patient was walking without pain.

Another clinical case consisted of a male patient in his 60s with disseminated non-small cell lung cancer who underwent an external beam radiotherapy followed by cementoplasty performed for bone metastases located on the spine and the right sacroiliac joint [53]. Two months after surgery, a fistula occurred, with clinical evidence of infection of the cement located in the right sacroiliac joint. Surgery was required to remove the cement and to debride and abscess. The patient developed catheter-related bacteremia due to ceftazidime-resistant *P. aeruginosa,* and he received IV imipenem/cilastatin. Despite antibiotic treatment, the patient still had a fever with purulent local secretion, and a CT scan revealed persistent osteomyelitis caused by XDR *P. aeruginosa* only susceptible to polymyxins and ceftolozane/tazobactam. As an alternative treatment, during the surgical procedure, debridement of the necrotic bone was performed, and a bacteriophage cocktail (10^8^ PFU/mL) was brought into contact with the bone in the cavity. The patient remained in ventral decubitus for 4 h to ensure that the phages remained in contact with the infected bone. As the patient had mild kidney injury, it was decided to use local administration of colistin. In addition, ceftolozane/tazobactam was given intravenously. At the time of surgical reconstruction, the macroscopic aspect was extremely favorable. After reconstruction, no bacteria grew in the culture and the healing was rapid.

Another scenario in which bacteriophage therapy has been widely used is in *P. aeruginosa* infections pre- and post-transplant. Nieuwenhuyse et al. described the case of a male toddler suffering from atresia with liver transplantation, with the nosocomial acquisition of extensively drug-resistant (XDR) *Pseudomonas aeruginosa* susceptible to colistin and intermediately susceptible to aztreonam [22]. The child presented multiple hepatic abscesses and severe septicemia. Despite intravenous (IV) antibiotic therapy, blood and abscess samples continued to grow XDR *P. aeruginosa*. Due to antibiotic therapy failure and the child’s critical situation, the decision was to initiate adjuvant phage therapy with bacteriophage cocktail BFC1. A phage cocktail (10^7^ PFU/mL) was administered intravenously combined with antibiotics (gentamycin, colistin, and high doses of aztreonam). Phage therapy combined with antibiotics controlled bloodstream infection and led to liver retransplantation after 72 days of combined treatment. More than two years after the second liver transplantation, total clearance of *P. aeruginosa* colonization was observed.

Law et al. described the case of a 26-year-old female with cystic fibrosis on the lung transplant waitlist with a pulmonary exacerbation leading to acute-on-chronic respiratory failure complicated by a pneumothorax [54]. She was colonized by two MDR *P. aeruginosa* strains: one non-mucoid susceptible to colistin and the other one mucoid susceptible to meropenem and piperacillin-tazobactam. She was treated with antibiotics for 4 weeks: the first two weeks with piperacillin-tazobactam, colistin, and azithromycin, and for the last two weeks, piperacillin-tazobactam was replaced by a carbapenem. At the end of the 4 weeks, the patient was transitioned to inhaled colistin. One week after discontinuation of IV antibiotics, the patient worsened and she was restarted on IV antibiotics (vancomycin, colistin, and meropenem, which were switched to piperacillin-tazobactam due to susceptibility profiles). Despite antibiotic treatment, the following week she experienced progressive respiratory and renal failure, attributed to colistin. At this time, they obtained approval for starting adjuvant phage therapy with AB-PA01, a cocktail of four bacteriophages. AB-PA01 was administered every 6 h (10^9^ PFU/mL) intravenously for 8 weeks. The patient received concomitant ciprofloxacin and piperacillin-tazobactam for 3 weeks. Finally, ciprofloxacin was discontinued, and doripenem was added based on updated susceptibility profiles. After the end of bacteriophage therapy, she did not have a recurrence of *P. aeruginosa* pneumonia and CF exacerbation. She underwent successful bilateral lung transplantation 9 months later.

Aslam et al. described the cases of two lung transplant recipients that received bacteriophage therapy for complicated MDR *P. aeruginosa* infections [55]. The first one was a 67-year-old man who underwent a bilateral transplant for hypersensitivity to pneumonitis, complicated by multiple episodes of *P. aeruginosa* pneumonia. He developed chronic lung allograft dysfunction and progressive kidney failure. The patient suffered two distinct episodes of MDR *P. aeruginosa* pneumonia that were treated with bacteriophage therapy along with concomitant antibiotics. For the first episode, he received a 2-week course of IV and nebulized AB-PA01 (10^9^ PFU/mL) as an adjunct to systemic antibiotics (piperacillin-tazobactam and colistin). After two weeks of treatment, he had significantly decreased inflammation and minimal respiratory secretions. Nebulized phage therapy was extended by an additional week without systemic antibiotics in an attempt to repopulate the airways with normal respiratory flora. By day 21 of treatment, bronchoalveolar lavage (BAL) cultures did not include *Pseudomonas* bacterial species, suggesting the reestablishment of respiratory flora. The patient completed inhaled phage therapy by day 29. However, on day 46, the patient suffered another episode characterized by clinical decompensation with respiratory failure and septic shock. In his respiratory cultures grew mucoid MDR *P. aeruginosa*; systemic antibiotics (piperacillin-tazobactam, tobramycin, and inhaled colistin) were restarted, and phage therapy was used again. In this case, bacteriophage therapy consisted of distinct courses of AB-PA01-m1 (prefixed cocktail of phages plus one new specific bacteriophage, 10^9^ PFU/mL) and Navy phage cocktail 1 (personalized phage cocktail, 10^9^ PFU/mL) with clinical resolution of pneumonia. After finishing treatment, the patient received suppressive bacteriophage therapy with Navy phage cocktail 1 and Navy phage cocktail 2 (10^9^ PFU/mL) from day 93 to day 150. During this period and the following 3 months, there was no active *P. aeruginosa* pneumonia. In another case, a 57-year-old woman with non-CF bronchiectasis colonized by MDR *P. aeruginosa* was only susceptible to colistin; she experienced significant bilateral airway ischemic injury, and developed recurrent MDR *P. aeruginosa* infections post-transplant. She also developed *Mycobacterium abscessus* infection, initially treated with imipenem, tigecycline, and inhaled colistin. As a result of nephrotoxic antibiotic treatment, she had progressive renal failure. Due to the inability to clear *P. aeruginosa* from respiratory cultures and concern that the infection was preventing airway healing, bacteriophage therapy was initiated. The patient was treated with a 4-week IV AB-PA01 and continued only with inhaled colistin concomitantly. The patient clinically responded to treatment, and no additional *P. aeruginosa* was cultured since the start of phage therapy until 60 days after completion. The isolate grown at day 60 and subsequent strains showed improved antibiotic susceptibility. Additional infections were successfully treated with piperacillin-tazobactam, and by day 90 she was discharged from the hospital.

Chen et al. reported the case of a 68-year-old man who suffered broncho-pleural fistula (BPF)-associated empyema and pneumonia caused by carbapenem-resistant *P. aeruginosa* [56]. The patient’s lung had been destroyed after tuberculosis and repeated hemoptysis for 2 years. A personalized lytic pathogen-specific two bacteriophage preparation was administered nebulized and injected intrapleurally to the patient continuously for 24 days in combination with conventional antibiotics IV (amikacin, azithromycin, imipenem, and ceftazidime-avibactam, among others). The combined treatment was well tolerated, resulting in clearance of the pathogen and improvement of clinical outcome.

Phage therapy has also been applied in the treatment of infections related to cardiothoracic surgery. A 76-year-old male patient with relapsing *P. aeruginosa* mediastinal and aortic graft infection was treated with moderately effective and indefinite IV ceftazidime [57]. The patient was an ideal candidate for bacteriophage therapy, so a procedure was proposed that comprised local administration of phage OMKO1 (10^7^ PFU/mL) and ceftazidime solution into the mediastinal fistula. The day after the procedure, the patient showed signs of improvement and was discharged from IV ceftazidime; the patient returned home shortly thereafter. Rubalskii et al. also reported critical infections related to cardiothoracic surgery in which bacteriophage therapy was necessary [58], such as a 13-year-old male patient with *P. aeruginosa*-infected thoracotomy wound after lung transplantation, not eradicated after conventional treatment. The patient received local administration of PA5 and PA10 (10^10^ PFU/mL) bacteriophages concomitantly with IV colistin and ceftazidime-avibactam. After bacteriophage–antibiotic treatment, the cardiothoracic wound fully healed, and *P. aeruginosa* was not detected again.

Finally, for bone-related infections, administration of antibiotics and phages concomitantly has been applied. Racenis et al. depicted the case of a 21-year-old patient with persistent MDR *P. aeruginosa* femur osteomyelitis, regardless of extensive antibiotic treatment and surgical procedures [23]. The combination of IV ceftazidime-avibactam and local administration of a phage cocktail (10^7^ PFU/mL) allowed for bacterial eradication and avoided leg amputation.

Tkhilaishvili et al. reported the case of an 80-year-old woman with metabolic syndrome (diabetes mellitus type 2, obesity, and hypertension), chronic kidney failure, diagnosis of relapsing right knee PJI, and chronic osteomyelitis of the femur after a gunshot injury [59]. One year earlier, the knee prosthesis was explanted, successfully treated, and reimplanted due to positive cultures of *Klebsiella pneumoniae* and *Providencia stuartii*. Three months after reimplantation, two morphologically distinct MDR *P. aeruginosa* isolates grew from the aspirated synovial fluid (one only susceptible to colistin and the other susceptible to ceftazidime and colistin). The knee prosthesis was explanted, and during surgery, an antibiotic-loaded cement spacer (containing 1 g gentamycin and 1 g clindamycin per 40 g poly(methyl methacrylate)) and four drainage tubes were placed. Adjunctive local bacteriophage therapy was applied during surgery (10^8^ PFU/mL), followed by administration every 8 h through the drain tubes as a delivery system for 5 days. Moreover, after surgery, intravenous treatment with colistin, meropenem, and ceftazidime was started. The drainage fluid was collected for culture before bacteriophage instillation on days three, four, and five of phage treatment, and no *P. aeruginosa* was isolated.

Sinner et al. recently reported the case of a 25-year-old male with exposed calvarium in the left parietal–temporal region, due to accidental electrocution burn wounds, complicated by the development of skull osteomyelitis caused by *P. aeruginosa* [60]. After the failure of traditional (debridement and antibiotic) treatment, Whole Genome Sequencing (WGS) revealed increased MICs of all available β-lactams (except cefiderocol), likely due of the presence of blaGES-1, a β-lactamase gene, in combination with MDR efflux pumps MexD and MexX, in all six of the patient’s isolates. After debridement of the infected scalp and bone, the patient was transitioned to cefiderocol but continued having relapses. Therefore, the patient received IV bacteriophage Pa14NPøPASA16 (1.7 × 10^11^ PFU) as adjuvant treatment for 6 weeks. The patient showed local wound improvement, with no further relapsing episodes and no abnormal laboratory values or findings on clinical exam suggesting toxicity. More than 12 months after ending antimicrobial treatment, the patient remained infection free.

## 5. Concluding Remarks

The worldwide spread of antibiotic resistance and the multiple failed antibiotic therapies against infectious diseases have made clear the urgent need to use an alternative or adjuvant to antibiotics. Phage therapy permits a specific union between the phage and the desired pathogen, becoming one of the most promising alternatives against infectious diseases produced by multi-drug resistant bacteria. The specificity of phages and the appearance of resistance against phages makes the use of cocktails more desirable in therapy, as shown in the in vivo and case-report studies. Despite the antibiotic–phage combination used in the mentioned case reports, not only against *P. aeruginosa* but also against most pathogens, there is a lack of in vivo studies with antibiotic–phage combinations. Interestingly, the scarce number of in vivo studies show a reduction of bacterial growth or eradication of the bacteria during and after phage therapy. The best antibiotic pairing should be chosen in consideration of the patient’s sensitivity and the clinical presentation. Although nebulized phage administration is showing successful and promising results. Though the majority of clinical cases applied an intravenous treatment, this does not mean that this is best method of administration. To answer this question, a clinical trial should be performed to measure phage concentration and antiphage antibodies over time using both nebulized and intravenous routes.

The efficiency of phage therapy is still intrinsically related to the specific case of the patient. As reviewed here, the single use of antibiotic therapy did not eradicate the infection; however, the combination between antibiotics and bacteriophage cocktails did show promising results, with total eradication of the infection and no further relapses for the patient in some cases. Moreover, in all cases, the administration of phages combined with antibiotics achieved an improvement in the clinical case or a decrease of the bacterial load.

In the case reports reviewed here, there was no toxicity associated with phage administration, and no abnormal laboratory results were obtained nor significant clinical findings in the patient post-treatment that would suggest toxicity derived from the phage therapy.

The combination of phages with antibiotics could be a realistic way to eradicate infections caused by MDR/XDR *P. aeruginosa* strains using a personalized therapy, although more in vivo studies are needed to analyze the limitations.

## Figures and Tables

**Table 1:** Summary of in vivo studies using phage–antibiotic combination against MDR *P. aeruginosa*.

Infection Model	Bacteria	Phage Therapy	Antibiotic Combination	Outcome	Reference
Lung infection, mouse	*P. aeruginosa* MDR	PEV20 (10^6^ PFU/mg)	Ciprofloxacin (0.33 mg)	Reduced bacterial load by 5.9 log	[45]
Acute immunocompromised, mouse	*P. aeruginosa* MDR	Three-phage cocktail (10^9^ PFU/mL)	Alone or with Meropenem	Enhanced therapeutic protection against pulmonary infection	[43]
Cystic fibrosis zebrafish	*P. aeruginosa* (PA01)	Four-phage cocktail (300–500 PFU/embryo)	Ciprofloxacin (100 µL)	Reduced embryos lethality	[44]
Dorsal wound, mouse	*P. aeruginosa* (PA01)	PAM2H cocktail (10^8^ PFU/mL)	Ceftazidime	Synergistic reduction in bacterial burden	[18]

**Table 2:** Summary of clinical case reports using phage–antibiotic combination against MDR *P. aeruginosa*.

Disease	Bacteria	Phage Therapy	Antibiotic Combination	Outcome	Reference
Prosthetic joint infection (PJI)	*P. aeruginosa*	Three-phage cocktail (10^9^ PFU/mL)	Ciprofloxacin Ceftazidime	Rapid improvement of patient’s health	[46]
Catheter-related bacteremia	Pandrug-resistant *P. aeruginosa*	Personalized three-phage cocktail (10^6^ PFU/mL) IV 3 h for 21 days	IV Cefiderocol 2 weeks later IV Colistin	Favorable to patient after 21 months follow-up	[47]
Catheter-related bacteremia	*P. aeruginosa* XDR	Phage cocktail (10^8^ PFU/mL) by direct contact with the infected bone for 4 h	Colistin (local) IV Ceftolozane/Tazobactam	Favorable, with no bacterial growth and rapid healing of bone	[48]
Liver infection	*P. aeruginosa* XDR	IV BFC1 cocktail (10^7^ PFU/mL)	IV Gentamycin, Colistin and Aztreonam	Controlled the bloodstream infection, and retransplantation was possible after 72 days	[22]
Cystic fibrosis	*P. aeruginosa* MDR	IV AB-PA01 (10^9^ PFU/mL) every 6 h for 8 weeks	Ciprofloxacin and Piperaciclin-tazobactam for 3 weeks; added Doripenem	No *P. aeruginosa* recurrence or CF exacerbation	[49]
Pneumonia	*P. aeruginosa* MDR	1) Nebulized AB-PA01 (10^9^ PFU/mL) for 2 weeks 2) AB-PA01-m1 and Navy-1 phage cocktail (10^9^ PFU/mL)	Piperaciclin-Tazobactam and Colistin	No active *P. aeruginosa* pneumonia after 3 months	[50]
Recurrent infections post-transplant	*P. aeruginosa* MDR	IV AB-PA01 for 4 weeks (10^6^ PFU/mL)	Inhaled Colistin Piperaciclin-Tazobactam from day 60 to 90	No additional *P. aeruginosa* was cultured	[50]
Pneumonia	Carbapenem-resistant *P. aeruginosa*	Personalized two-phage cocktail preparations (10^8^ PFU/mL). Nebulized administration and intrapleural for 24 days	IV Amikacin, Azhitromycin, Imipenem, and Ceftazidime-Avibactam	Clearance of the pathogen and clinical improvement	[51]
Graft infection, bacteremia	*P. aeruginosa*	OMK01 (10^7^ PFU/mL)	Ceftazidime	General clinical improvement	[52]
Wound infection	*P. aeruginosa*	PA5 and PA10 (10^10^ PFU/mL)	IV Ceftazidime-Avibactam and Colistin	The wound completely healed, with no *P. aeruginosa* detection	[53]
Relapsing bacteremia	*P. aeruginosa* MDR	Local application of BFC 1.10 (10^7^ PFU/mL) cocktail	IV Ceftazidime-Avibactam	Bacterial eradication	[23]
Bacteremia	*P. aeruginosa* MDR	Local application (10^8^ PFU/mL) during surgery every 8 h for 5 days	IV Colistin, Meropenem, and Ceftazidime	No *P. aeruginosa* detection	[54]
